# PatchSorter: a high throughput deep learning digital pathology tool for object labeling

**DOI:** 10.1038/s41746-024-01150-4

**Published:** 2024-06-20

**Authors:** Cédric Walker, Tasneem Talawalla, Robert Toth, Akhil Ambekar, Kien Rea, Oswin Chamian, Fan Fan, Sabina Berezowska, Sven Rottenberg, Anant Madabhushi, Marie Maillard, Laura Barisoni, Hugo Mark Horlings, Andrew Janowczyk

**Affiliations:** 1 Institute of Animal Pathology, Vetsuisse Faculty, University of Bern, Bern, Switzerland; 2 Graduate School for Cellular and Biomedical Sciences, University of Bern, Bern, Switzerland; 3 Department of Biomedical Engineering, Case Western Reserve University, Cleveland, OH USA; 4 Toth Technology LLC, Toth Technology LLC, New Brunswick, NJ USA; 5 Department of Pathology, Division of AI & Computational Pathology, Duke University, Durham, NC USA; 6 AI Health, Duke University, Durham, NC USA; 7 Institute of Pathology, Lausanne University Hospital, Lausanne, Switzerland; 8 Bern Center for Precision Medicine, University of Bern, Bern, Switzerland; 9 Department of Biomedical Engineering, Emory University and Georgia Institute of Technology, Atlanta, GA USA; 10 Atlanta Veterans Medical Center, Atlanta, GA USA; 11 Department of Medicine, Division of Nephrology, Duke University, Durham, NC USA; 12 Department of Pathology, The Netherlands Cancer Institute, Amsterdam, The Netherlands; 13 Department of Oncology, Division of Precision Oncology, Geneva University Hospitals, Geneva, Switzerland; 14 Department of Diagnostics, Division of Clinical Pathology, Geneva University Hospitals, Geneva, Switzerland

**Keywords:** Image processing, Machine learning, Biomarkers

## Abstract

The discovery of patterns associated with diagnosis, prognosis, and therapy response in digital pathology images often requires intractable labeling of large quantities of histological objects. Here we release an open-source labeling tool, PatchSorter, which integrates deep learning with an intuitive web interface. Using >100,000 objects, we demonstrate a >7x improvement in labels per second over unaided labeling, with minimal impact on labeling accuracy, thus enabling high-throughput labeling of large datasets.

The increasing digitization of routine clinical histology slides into whole slide images (WSI) has spurred great interest in the development of WSI-based biomarkers for diagnosis, prognosis, and therapy response^1–3^. These biomarkers are typically based on patterns associated with the location and type of individual histologic objects (e.g., cells—lymphocytes/epithelial; glomeruli—globally sclerotic (GS)/non-sclerotic (non-GS/SS)/segmentally sclerotic (SS); tubules—distal/proximal; tumor buds—present/absent). While current hardware and machine learning algorithms can locate and type objects at scale, the manual assignment and review of large labeled datasets used to train or validate models remains arduous. For example, a single WSI may contain over 1 million cells, which, if requiring a modest 1 second per cell to label, would result in ~12 non-stop days of effort. To aid experts (e.g., pathologists) in this labeling process, several image analysis algorithms have been proposed^4–9^. However, these algorithms tend to either (a) not be integrated into polished, user-friendly tools, making them unsuitable for usage by domain experts, or (b) are of a closed source, for-profit nature, creating a barrier to their broad usage, which potentially limits their continuous improvement via the facile integration and evaluation of new algorithms^10^ (Supplementary Table 1).

Appreciating the need for an open-source force multiplier for labeling histological objects, we here describe and make available to the community PatchSorter (PS). PS is a user-friendly, browser-based tool, which allows the user to leverage deep learning (DL) to quickly review and apply labels at a group, as opposed to a single object, level (Fig. 1). We demonstrated that this “bulk” labeling approach improves labeling efficiency across four use cases, spanning three levels of increasing object complexity (i.e., objects comprised of increasing number of cells and cell types) (Table 1).

**Fig. 1:**
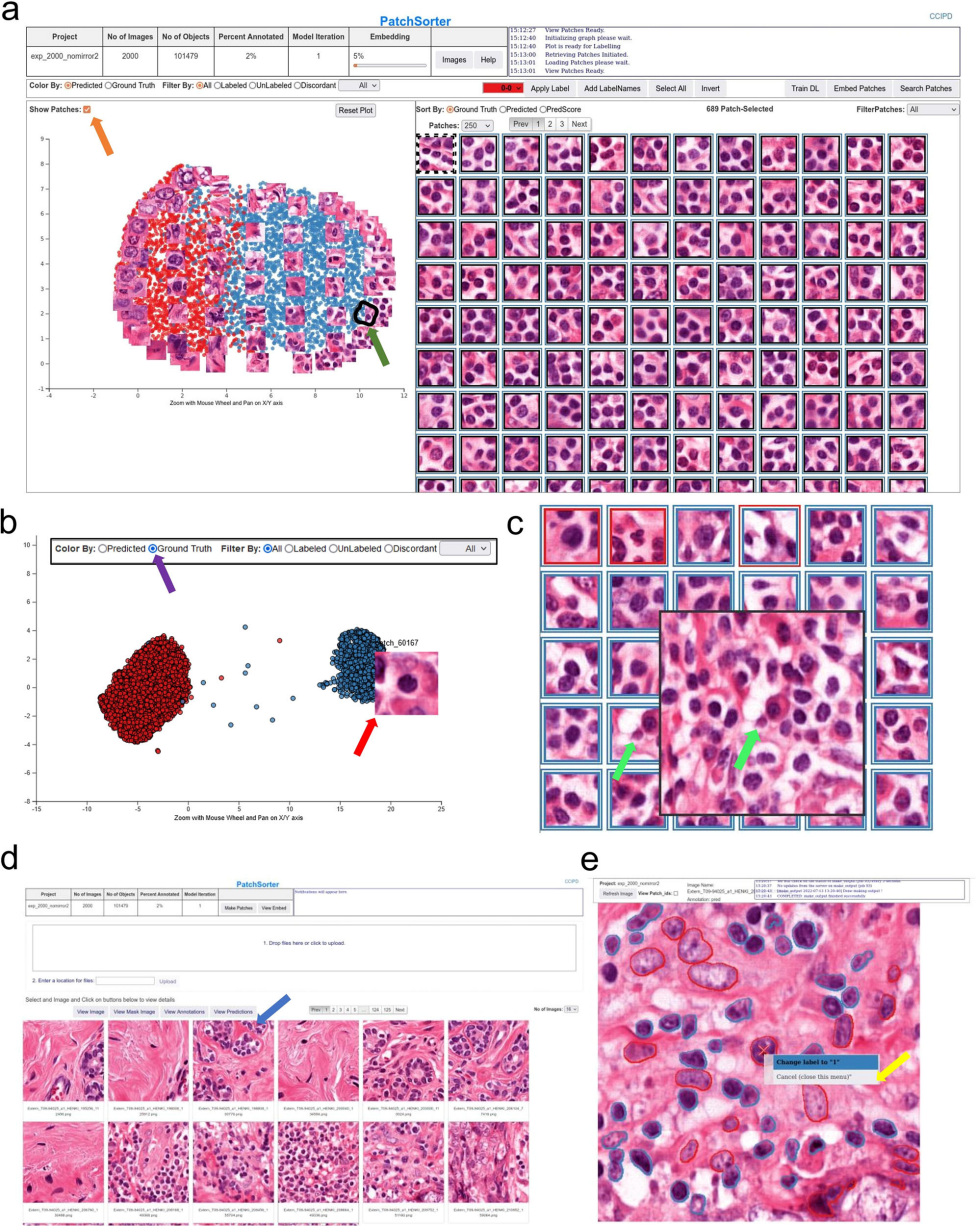
PatchSorter user interface. **a** The embedding plot after initial embedding (left) with the corresponding grid plot (right). The two-dimensional embedding plot places patches with the same deep-learned features in close proximity, causing objects with the same object class to cluster. The user lassos points (black contour with green arrow), which then appear in the grid plot for labeling using efficient keyboard shortcuts. In the embedding plot, a subset of patches can be overlaid to aid in selecting regions in the embedding space (orange arrow). **b** The embedding plot allows for coloring patches by prediction and ground truth (purple arrow). The embedding plot shows the same dataset as (**a**) after eight model iterations where the embedding space is well separated by ground truth labels. Hovering over a point in the embedding space shows the corresponding patch (red arrow). **c** Grid plot coloring shows current predictions and ground truth. The inner square color represents ground truth while the outer square color represents model prediction, with black indicating that the patch is not yet labeled. Right-clicking on a patch in the grid plot shows a larger region of interest (ROI) for context (green arrows). **d** From the image pane, prediction and ground truth labels can be visualized (blue arrow) in the output reviewer. **e** Here, object labels can be updated via a right click on the object (yellow arrow).

**Table 1:** Overview of use cases and measured efficiency improvements

Level of cellular and structural complexity	Histological primitive	Number of ROI	Number of histological objects labeled	PS total time (s)	PS human time (s)	PS efficiency total time (LPS)	PS efficiency human time (LPS)	Manual efficiency (LPS)	Speed up (θ)	PS efficiency lower bound (LPS) with speed up	PS efficiency upper bound (LPS) with speed up	Stain type	Concordance
Low	Breast cancer **nuclei:** lymphocytes vs. non-lymphocytes	2000	101479	235998	32735	1.92	3.1	0.43	7.21x	0.35 (0.81x)	9.6 (22.3x)	H&E	86%
Medium	Lung cancer **tumor-budding**: present vs. absent	27	1631	2471	1800	0.66	0.906	0.292	3.1x	0.49 (1.68x)	1.06 (3.6x)	H&E	93%
Medium	Kidney **tubules**: distal vs. proximal vs. abnormal	216	2298	10943	3648	0.21	0.63	0.218	2.89x	0.52 (2.47x)	0.95 (4.5x)	PAS	97%
High	Kidney **glomeruli:** SS vs. GS vs non-SS/GS	16158	16158	23978	20171	0.674	0.801	0.159	5.03x	0.57 (3.58x)	1.15 (7.23x)	PAS	96%

Description of the datasets used for validating PatchSorter along with the demonstrated efficiency gains in terms of labels per second (LPS) and concordance with an unaided approach. The difference between human time and total time is the inclusion of model training and embedding in the lableling time in total time, while it is removed for human time, as the human reader can be dismissed to perform other non-labeling related tasks. Manual efficiency (LPS_M_) for the same task is estimated based on the extrapolatation of manual labeling of a subset of the data within a 15-min interval. Upper and lower bounds for the efficiency of PS (LPS_PS_) are estimated within a 15-min interval sliding window with a 5-min interval stride (see Fig. 2). This creates robust estimates of the upper and lower bounds by smoothing potential outliers, as well as allowing for more accurate comparisons to LPS_M_. For the nuclei use case, speed increases of up to 22.3x (9.6 LPS) are observable while only being slightly slower than manual labeling in one of the measured 15-min intervals. For tubules, tumor buds, and glomeruli, PatchSorter offers a speed increase over manual labeling efforts, even for worst-case estimates.

*SS* segmentally sclerotic, *GS* globally sclerotic, *non-SS/GS* non-sclerotic.

PS enables labeling speed improvements by using DL-derived features to embed patches containing the object of interest (e.g., glomeruli) into a two-dimensional embedding space, such that similarly presenting objects are proximally located. The user then reviews patches within a localized region that are likely to correspond to the same class, thus enabling the assignment of labels in bulk (i.e., assignment of the same label to multiple objects at once) with increased efficiency. The DL model and associated embedding space is then iteratively refined with the user’s feedback, yielding improved class separability, further improving subsequent labeling efficiency (Supplementary Figs. 1,2).

To evaluate this improved efficiency, a labels per second (LPS) metric was compared between PS and an unaided approach, Quick Reviewer (QR, see Methods)^11^, across four use cases (Table 1, see Methods) totaling over 120,000 objects. QR was used to label a random subset of the data to estimate manual LPS (LPS_M_) per use case. Efficiency improvement was measured as the ratio (θ) between PS’s LPS (LPS_PS_) and LPS_M_. To ensure labeling efficiency improvements did not come at the cost of label fidelity, concordance between QR and PS-assigned labels was measured. Labeling for all use-cases was conducted by board-certified pathologists, after having received an introduction to the PS and QR user interfaces.

These results indicate that (a) PS provides sizable efficiency improvements in labeling objects of all levels of cellular and structural complexity, while (b) not coming at the cost of a loss of labeling accuracy (Table 1). Interestingly, differences remain in labels generated via PS and QR. This difference can be at least partially attributed to label uncertainty related to ambiguous objects, wherein labeling is likely to suffer from inter/intra-observer variability (Supplementary Figs. 3–6).

The usage of PS appears to proceed in two distinct workflows: (a) rapid bulk labeling on the periphery of the embedding space where objects with more obvious labels tend to be grouped and (b) slower intricate labeling at the interface between classes where object labels tend to be more challenging to determine. Notably, these challenging data points often drive improved class separation. As such, our suggested best practice is to alternate between the two workflows: (1) when class separation is high in the embedding plot, the operator should focus on bulk labeling, while (2) if class separation is low, labeling should be performed at the interface between classes. This interface labeling should result in improved class separation in the next embedding iteration, thus facilitating again bulk labeling (Supplementary Fig. 1).

The transition point between these two workflows appears to be use-case specific (Fig. 2). While in the nuclei use-case labeling speed improves with DL training, in the glomerular use case, a more time-consuming careful evaluation is required throughout the task, due to the difficult nature of differentiating between transitioning classes (e.g., SS with small areas of scarring mimicking non-GS/SS or with extensive segmental sclerosis mimicking GS).

**Fig. 2:**
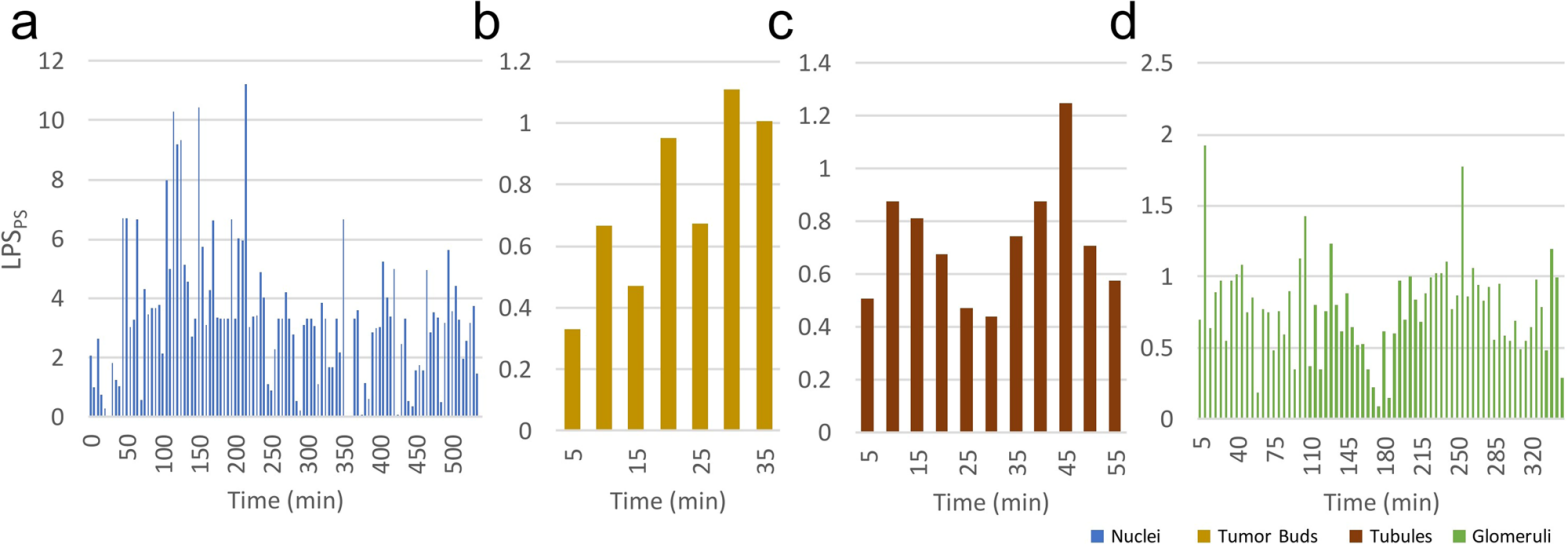
Time-dependent variability in labeling speed across different use cases. Efficiency metric LPS_PS_ over time measured in 5-minute intervals visualizing the time-dependent variability in labeling speed of PS for the **a** nuclei, **b** tumor bud, **c** tubules, and **d** glomeruli use case. The x-axis is the human annotation time in minutes and the y-axis is the labeling speed per second for a given time interval. Labeling performance over time varies per use case. For **a** nuclei labeling, a consistent performance increase over time is noted, consistent with the observed increase in class separation in the embedding space, as more labels were available to the model. As the entire dataset is labeled, performance decreased as easy-to-discern object labels were exhausted. For **b** tumor bud candidates, initial labeling efficiency was only marginally higher than manual baseline LPS. As more objects were labeled over time, labeling efficiency increased. For **c** tubule labeling, the initial embedding allowed for bulk annotation. In subsequent iterations, class separation decreased due to changes to the initially assigned labels and the imbalanced labeling of the four classes during the initial labeling phase. However, the addition of more object labels over time improved class separability and led to an increase in labeling efficiency in later iterations. Lastly, for **d** glomeruli labeling, the initial embedding allowed for bulk annotation of non-SS/GS, GS, and SS at the edge of the embedding plot, while later, nuanced labeling had to be employed due to the task’s difficulty.

From a usage perspective, after PS installation, no internet connection is required, enabling its use in clinical environments where data may not be anonymized. PS can be installed locally on commodity desktops or deployed on servers for remote access by experts (i.e., bringing the expert to the data), as datasets become too large to quickly transfer and clinical environments further restrict the installation of third-party software. While PS has been validated in this study on hematoxylin and eosin (H&E) and periodic acid-Schiff (PAS) staining, given the DL-based back end, PS can be considered agnostic to stain type and be used with any stain, image, or object type.

In conclusion, PS is a user-friendly, high-throughput object labeling tool being publicly released for community usage, review, and feedback. PS has demonstrated significant improvement in efficiency in object labeling in the hands of domain experts without sacrificing labeling accuracy. The source code of PS is freely available for use, modification, and contribution at www.patchsorter.com.

## Methods

### PatchSorter workflow

PS abstracts the concept of use cases by allowing the user to create a separate project per use case in a single PS instance. PS manages projects as fully independent entities, keeping track of project-specific model weights, images, and object labels. This added level of abstraction also has the advantage that it creates a unified PS workflow (see Supplementary Fig. 2) on a project and, therefore, also use case level. For each use case then, following the PS workflow, images containing regions of interest (ROIs) from multiple WSI were uploaded to PS together with a corresponding segmentation mask highlighting object location. PS then extracts patches, with user-configurable patch sizes, around the center of these objects to create an internal database for high-speed training. While a number of different self-supervised approaches are supported by PS (e.g., BarlowTwins^12^ and AutoEncoder^13^), a SimCLR^14^ using a ResNet18^15^ backbone was trained using contrastive loss, creating a use-case-specific DL feature space. Feature vectors are computed for each patch using this learned feature space, and are subsequently embedded using uniform manifold approximation and projection (UMAP)^16^ into two dimensions. As a result of this process, objects which look the same tend to be plotted near each other in the embedding plot. This allows the user to lasso regions on the embedding plot and provide the label for the selection in the grid plot (Fig. 1a). As more objects are labeled, PS is increasingly able to learn a more discriminative feature space for the categories of the specific task, by fine-tuning the self-supervised DL model on the newly provided labels in a semi-supervised fashion via the inclusion of an additional cross-entropy supervised loss function. As a result, subsequent iterations should demonstrate improved localized clustering “purity” (i.e., objects in the same cluster have the same label). This approach has two consequences, (a) the user can avoid intractably manipulating individual objects and instead provide bulk annotations to groups of objects with a single input, and (b) as the DL model (and thus the embedding space) is refined with the user’s feedback, the user can begin to see regions in the 2d space, where the underlying model is struggling to differentiate between class-types. The visibility of such regions affords the user the opportunity to better invest their time in selecting objects that, when labeled are most likely to further improve class separability in the next iteration, which in turn further improves subsequent labeling efficiency.

To facilitate the efficiency of this bulk labeling process, features from modern operating systems were implemented, such as drag-select and numerous intuitive keyboard shortcuts for (a) selecting all objects, (b) inverting the selection, as well as (c) changing the desired label (e.g., “1” selects the first class). If specific objects of interest are sought, PS provides content-based image retrieval, wherein the user may upload a patch of the object of interest, and similar objects from the dataset will appear for labeling within the standard workflow. PS was designed in a decoupled, modular, manner such that its backend technologies can easily be exchanged to evaluate different DL technologies, with minimal modifications to the base application. To ease integration with other workflows and pipelines, the output of PS is highly portable: mask images with color indicating class membership (Supplementary Fig. 1d). For more advanced users, the internal database can be directly employed in common downstream tasks, such as training large custom DL models. It is important to note, that the user retains full control over the accuracy of object labels at all times, and only confirmed labels are stored. Usefully, these newly generated ground truth labels (as well as predicted labels), can be visualized through PS for rapid tile-level review, wherein individual object labels may still be modified as needed (Fig. 1e).

### Manual unaided baseline efficiency estimation

Quick Reviewer (QR)^11^, an open-source object labeling tool, was employed as the unaided baseline approach for comparison against PS. QR is a simple web-based framework which presents an image patch to the user, one at a time, and collects their label determination via a button click. It should be noted that QR already offers notable efficiency advantages over true unaided manual object labeling pipelines, as objects are directly presented to the user, which obviates the time-consuming process of (a) finding specific objects in WSIs and (b) transitioning between different WSIs. As such, QR times can be considered optimistic as compared to a “fully” unaided approach, which are increasingly becoming less common in practice.

### Metrics for evaluating PS efficiency improvement

For comparing PS to QR we introduce a labels per second (LPS) metric. For each of the 4 use cases described below, QR was used to label a random subset of the data to estimate LPS and extrapolate manual LPS (LPS_M_) for the entire dataset. For PS, we measure LPS in total time and human time (LPS_PS_). The difference between human time and total time is the inclusion of model training and patch embedding in total time, while it is removed for human time, as the human reader can be dismissed to perform other non-labeling related tasks. Efficiency improvement is then measured as the ratio (θ) between LPS_PS_ and LPS_M_. To ensure these labeling efficiency improvements did not come at the cost of unacceptable fidelity loss, the subset of data manually labeled is quantitively compared using the concordance metric to the labels produced via PS. Given a set of objects labeled in both QR and PS, we measure the concordance metric as the percentage of objects in the set with label agreement in PS and QR (i.e., accuracy measure in multiclass classification). To preclude the potential effects of inter-observer variability on label concordance, object labeling for both QR and PS in a use case was conducted by the same pathologist.

### Use case 1: nuclei labeling in triple-negative breast cancer

Tumor-infiltrating lymphocytes (TILs) have emerged as a biomarker of interest in breast cancer, with mounting evidence of their prognostic and predictive value in triple-negative breast cancer^17^. TILs are labeled in accordance with the immune-oncology working group guidelines for immune infiltration scoring in breast cancer^18^ into lymphocyte and non-lymphocyte.

To begin, 2000 1000 × 1000 pixel image tiles were randomly cropped from *n* = 21 fully deidentified H&E WSIs scanned at 40x Magnification from the MATADOR^19^ cohort, ensuring sufficient quality (e.g., exclusion of tissue folds or blurry regions). ROIs were stain normalized based on a reference tile from the MATADOR^19^ cohort using the Vahadane stain normalization^20^ implementation from StainTools (https://github.com/Peter554/StainTools). Using the HoverNet^21^ implementation from histocartography ^22^, nuclei were segmented to provide the object location information to PS. Following the PS workflow (Supplementary Fig. 2), ROIs and corresponding object segmentation mask were uploaded into PS where nuclei were extracted from the ROI into 64 × 64-pixel patches with the nuclei centered.

For the QR experiment, an additional label was included to capture patches where no nucleus is present in the patch center due to nuclei segmentation errors. As in this use case, the user was forced to label the whole cohort, and a decision for every patch had to be reached. In use cases which employ large-scale automatic object detection, the inclusion of a general negative “non-object” class in PS might be worth considering. The concordance metric was calculated only on objects with corresponding PS labels. Labeling of the nuclei was conducted by H.M.H. for both PS and QR.

### Use case 2: detection of tumor budding in pulmonary squamous cell carcinoma

Tumor buds, defined as clusters of cancer cells composed of fewer than five cells^23^, is an invasive pattern that has been described in solid tumors (e.g., colon cancer). Tumor budding has attracted interest as a prognostic biomarker in lung cancer, with the presence of tumor buds being associated with worse patient outcomes.

Here, 27 2000 × 2000 pixel ROIs were extracted at 40x from *n* = 3 fully deidentified H&E stained lung cancer samples. A u-net^24^ model was applied to each ROI to segment potential tumor bud candidates for further labeling into absent/present. ROIs were stain normalized using the Vahadane stain normalization^20^ method implemented in StainTools and each ROI was downsampled to 500 by 500 pixel using nearest-neighbor interpolation. 1761 tumor bud candidates were extracted into 64 × 64-pixel patches by PS with a single potential tumor bud centered.

Small changes to the PS user interface were made to show a larger 256 × 256 image instead of the 64 × 64 image used for training the DL model. This provided additional context was requested by the reader to improve their decision-making comfort; these changes are available in the PS code repository. In QR, patches were presented with an overlay of the u-net segmentation mask for indicating tumor bud position in the ROI, as multiple tumor buds might be present in the ROI.

In addition to absent/present, PS and QR were set-up to include an “unsure” label, allowing for the labeling of patches where the pathologist was not comfortable in making a definitive decision during the experiment. The reported accuracy is measured between all labels present in QR and PS (absent/present/unsure).

Discussion of the discordant cases between QR and PS indicated that the additional context provided by QR led the pathologist to be less confident in labeling patches as “absent”, while in PS, patch similarities to other “absent” examples in the embedding space led the pathologist to more likely label these patches as ‘absent’ (Supplementary Fig. 4). Therefore, the user-perceived agreement between PS and QR is likely higher than the concordance score indicates. Tumor buds were labeled by M.M. for both PS and QR.

### Use case 3: renal tubular classification

Tubules are a major component of the nephron, the functional unit of the kidney. The two major types of tubules in the kidney cortex are the proximal and distal tubules, and they are vulnerable to a variety of injuries across diseases (e.g., atrophy, acute injury, osmotic changes, etc.). For this use case, tubules were labeled into four classes: proximal, distal, abnormal, and other (i.e., false positive from the a priori tubule segmentation step and collecting ducts or thin limb of loop of Henly tubules in the medulla)^25^.

About 216 ROIs were extracted from fully deidentified WSI from the NEPTUNE^26^ PAS WSI cohort at 20x Magnification and uploaded into PS. ROIs were stain normalized using the Vahadane stain normalization^20^ implementation from StainTools. 10,129 Tubules were extracted into 256 by 256-pixel patches with a single tubule centered based on tubule annotations created in QuPath^27^. Finally, a subset of 2298 tubules were labeled by L.B. using PS to estimate labeling efficiency.

### Use case 4: renal glomerular classification

Glomeruli, the filtration organelles of the kidney nephrons, can undergo a variety of morphologic changes. For this use case, we selected diseases where glomeruli can undergo segmental to global scarring. Glomeruli were labeled into five categories: globally sclerotic (GS), segmentally sclerotic (SS), non-sclerotic glomeruli (non-SS/GS), non-glomeruli (i.e., false positive from a priori glomeruli segmentation step) and uncertain (i.e., distinction between SS and GS is challenging by visual inspection)^28,29^. The high complexity of these organelles consisting of various cell types, a capillary tuft, a mesangial stalk, a urinary space, and a capsule, and the high heterogeneity in image presentation of GS and SS glomeruli, allows for the showcasing PS’s ability to provide improved labeling efficiency of complex objects. The reported accuracy is measured between GS, SS, non-GS/SS, and non-glomeruli labels. Cases labeled as uncertain were excluded as their ambiguous nature would not lead to meaningful conclusions regarding the concordance between PS and QR.

For the experiment, 16,158 glomeruli from 241 fully deidentified NEPTUNE^26^ and CureGN^30^ PAS WSIs were used. Glomeruli were previously manually segmented using QuPath^27^ and preprocessed into 256 by 256-pixel ROIs extracted at 40x magnification, each containing a singular glomerulus centered in the ROI. ROIs were normalized using Vahadane stain normalization^20^ using the StainTools library. ROIs and corresponding segmentation masks were uploaded into PS according to the PS workflow (Supplementary Fig. 2). Patches were created using the full ROI. Glomeruli were labeled by L.B. for both PS and QR.

### Configuration and hyperparameters

The default version of PS is nearly fully configured. The few hyperparameters of interest are easily modifiable through the configuration file. In the use cases discussed here, the hyperparameters requiring change relate to the patch size extracting the objects from the ROI images as well as the encoder size of the DL model, governing how much information for a given patch can be used by the model to assess patch similarities. Patch size was chosen based on object size and magnification, such that each object is fully visible in a patch. In the use cases presented (see Table 1), the encoder size was set equal to the patch size. For example, in the glomeruli classification use case, the patch size was configured as 256× 256 pixels, with the encoder size being configured as 256. This parameter-setting approach appears to yield a sufficient starting point for using PS efficiently.

### Experiment setup

Each experiment was conducted on an Ubuntu Server 20.04LTS equipped with a Nvidia GeForce RTX 2080 Ti.

### Reporting summary

Further information on research design is available in the Nature Research Reporting Summary linked to this article.

### Supplementary information

Supplementary information

Reporting Summary

## Data Availability

Data requests should be forwarded to the corresponding authors of the cited sources.

## Code availability

All used software libraries and custom software are listed at https://github.com/choosehappy/PatchSorter.
