# Supplementary material for: PatchSorter: a high throughput deep learning digital pathology tool for object labeling
Source: NPJ Digit Med. 2024 Jun 20;7:164. doi: 10.1038/s41746-024-01150-4 (PMC11190251; doi:10.1038/s41746-024-01150-4)
Supplement: Supplementary file 1 — Supplementary information [file 41746_2024_1150_MOESM1_ESM.pdf]

Supplementary Tables

Supplementary Table 1. Overview of image analysis algorithms for aiding in object labeling objectives.

**Study**	**Keywords**	**Method**	**Availability**
**PatchSorter** (ours)	Human-in-the-loop<br>Active learning<br>Self-supervised learning<br>Interactive embedding	Fully integrated web-based user interface for continual exploration of the object embedding space, deep learning model training and object labeling.	Open source with BSD-3 clause license
Pati et al.[4]	Limited annotations<br>Co-representation learning	Reducing the number of annotations needed in classification tasks through co-representation learning.	Code not available
Bengar et al.[5]	Human-in-the-loop<br>Active learning<br>Self-supervised learning<br>Sample retrieval	Heuristic to select the optimal subset of unlabeled examples to present to the oracle (e.g., human expert) based on the set of prior labeled objects. The active learning loop concludes by adding the labels of the selected examples to the set of labeled objects.	Code not available
Menon et al.[6]	Human-in-the-loop<br>Active learning<br>Sample retrieval	Heuristic to select the optimal subset of unlabeled examples to present to the oracle (e.g., human expert) based on a set of prior labeled objects. The active learning loop concludes by adding the labels of the selected examples to the set of labeled objects.	Code not available
Holub et al.[7]	Human-in-the-loop<br>Active Learning<br>Sample retrieval	Heuristic to select the optimal subset of unlabeled examples to present to the oracle (e.g., human expert) based on a set of prior labeled objects. The active learning loop concludes by adding the labels of the selected examples to the set of labeled objects.	Code not available
Lutnik et al.[8]	Human-in-the-loop<br>Active learning	Command line tool to convert annotations between Aperio ImageScope and python to facilitate an active learning loop. Import of model output to ImageScope allows for review and correction of deep learning output for further training. While PatchSorter integrates a web-based user interface directly, Lutnik et al. employ ImageScope, which is a) proprietary, b) only locally installable, and c) necessitates switching between the user interface and command line interface during the active learning loop.	Open source/ proprietary
Das et al.[9]	Active Learning<br>Sample retrieval	Heuristic to select the optimal subset of unlabeled examples to present to the oracle (e.g., human expert) based on a set of prior labeled objects. MATLAB based implementation available to calculate the heuristic. The available implementation is not licensed for open-source use and does not include a user-interface or a documented command line interface.	Available but not open-source licensed
LindvaN et al.[10]	Interactive annotation<br>Superpixel	Superpixel based interactive tissue annotation tool developed by Sectra AB.	Proprietary

Supplementary Figures

Supplementary Figure 1. PS enables direct workflow from object labeling to downstream analysis.

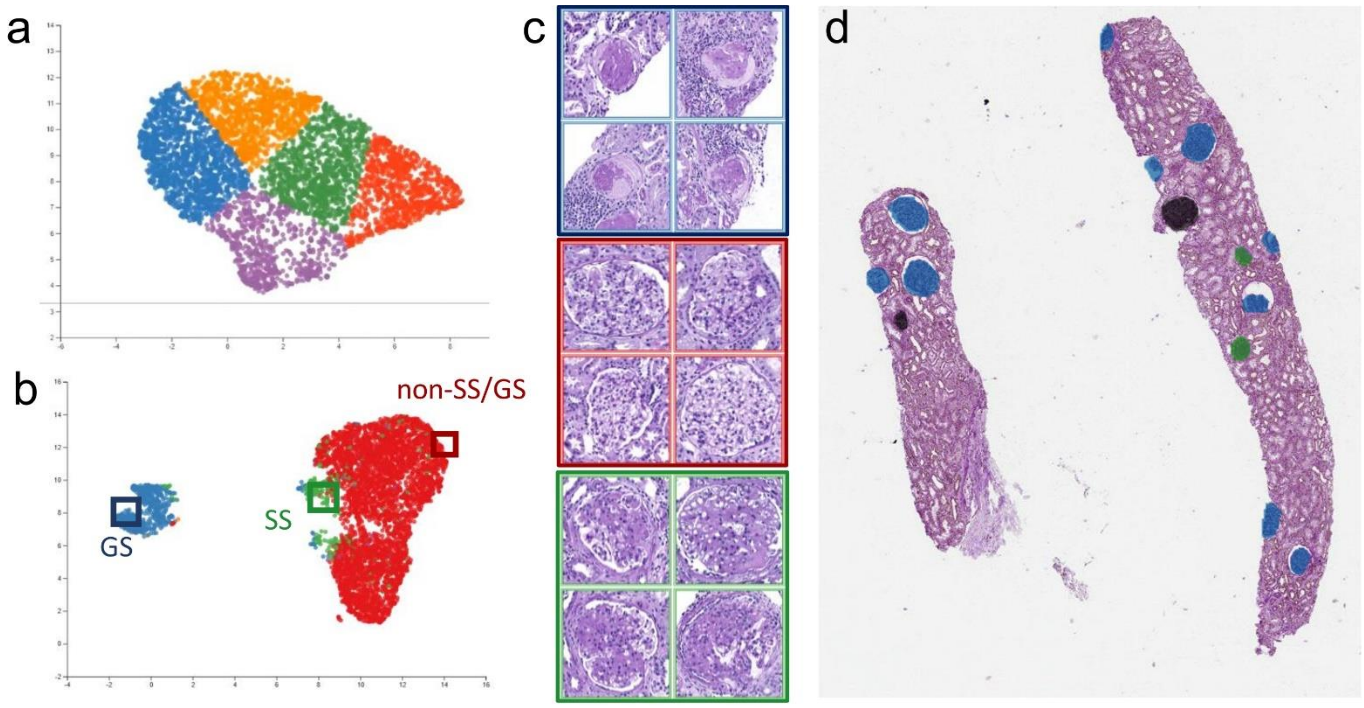

Supplementary Figure 1. a Initial unsupervised embedding space of the glomeruli classification use-case clusters the embedding space equally amongst the 5 classes. **b** After four training, embedding and labeling cycles, the embedding space shows clear class separation (blue, green, and red box), **c** yielding high class purity regions suitable for bulk labeling. **d** As PS supports the extraction of objects from ROIs, if suitable ROIs are supplied, PS can generate ground truth and prediction masks directly usable for downstream analysis.

Supplementary Figure 2. PS workflow diagram

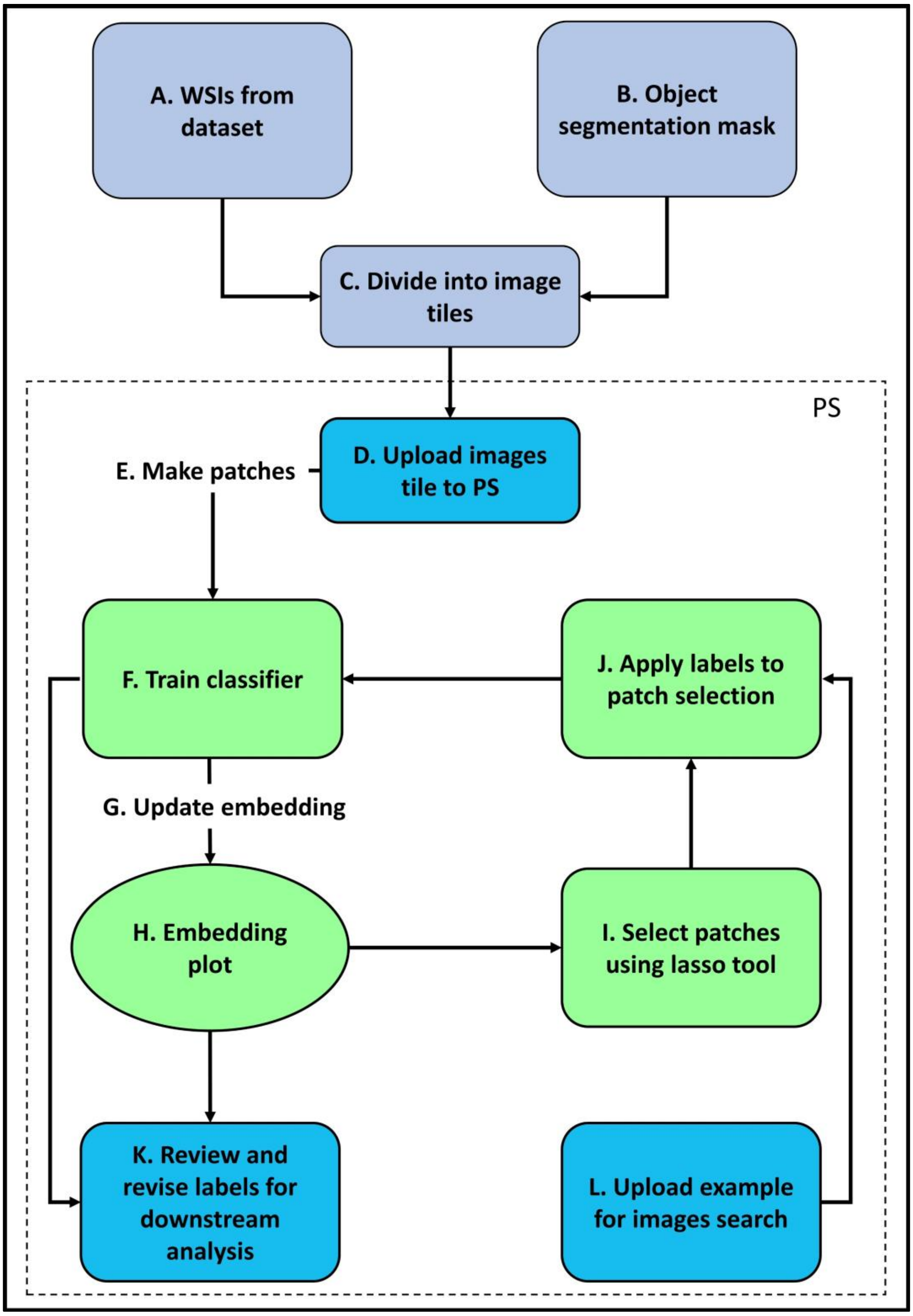

Supplementary Figure 2. Flowchart illustrating the workflow of PS. (A & B & C) WSI are divided into tiles for uploading into PS according to the use case specific workflow (see **Methods**). (D) Images tiles are uploaded into PS where (E) tiles are then subsequently divided into smaller patches of use case specific size (Table S1) with the objects centered suitable for the deep learning model. (F) Patches are then used to train a deep learning model to encode patch information into a feature vector. (G) Each patch is subsequently embedded into a two-dimensional space using UMAP from the feature representation layer of the deep learning model. (H->I) From this the iterative cycle begins where the user selects patches from the UMAP embedding plot (Figure 1a) and assigns class labels to the selection (Figure 1a). Label information is incorporated into the DL training which increased class separation in the embedding space. (K) At any point, model predictions can be reviewed on an ROI level (Figure 1bc) and object labels can be revised directly on the output generation (Figure 1c), which can be used for further downstream analysis. (L) Instead of the lasso tool (H), the user can alternatively select patches from the embedding space using a reference image.

Supplementary Figure 3. Discordant cases in nuclei labeling in triple-negative breast cancer use case

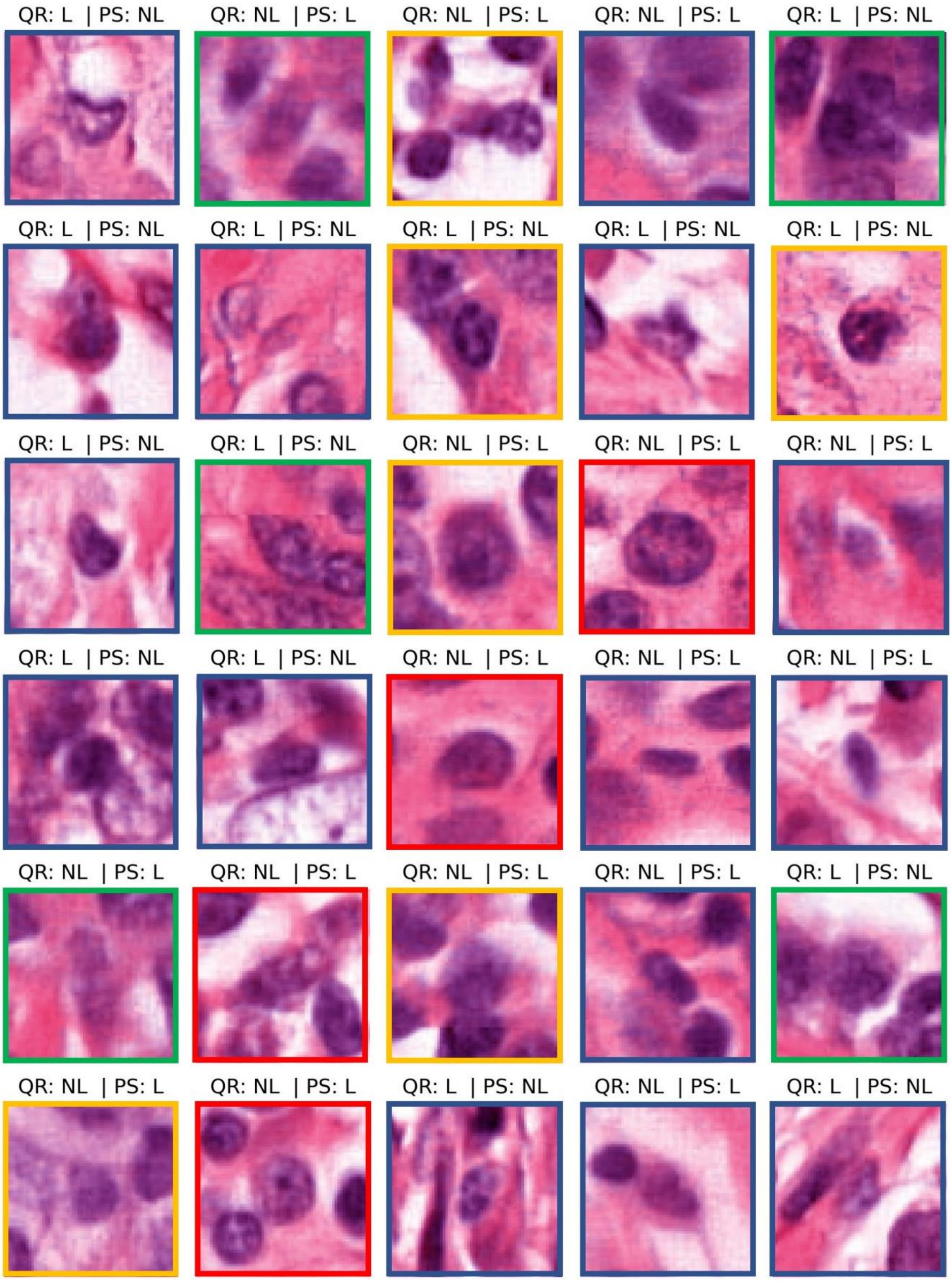

Supplementary Figure 3. Discordant cases between PS and QR in the nuclei use-case of with patches labeled as lymphocyte (L) or non-lymphocyte (NL). Discordant cases can be broadly distinguished into four categories. First, difficult exemplars with inter and intra observer variability (blue box). Second, as the combined human labeling time exceeded 9 hours, human error is likely to occur for some patches (red box). Third, label is context dependent (yellow box). As QR shows the whole ROI at once, context is immediately available, while in PS context information can be invoked manually per patch as deemed necessary by the user. Fourth, label is not accurately assignable due to either multiple cells present at the center of the patch or insufficient image quality (green box).

Supplementary Figure 4. Discordant cases in detection of tumor budding in pulmonary squamous cell carcinoma use case

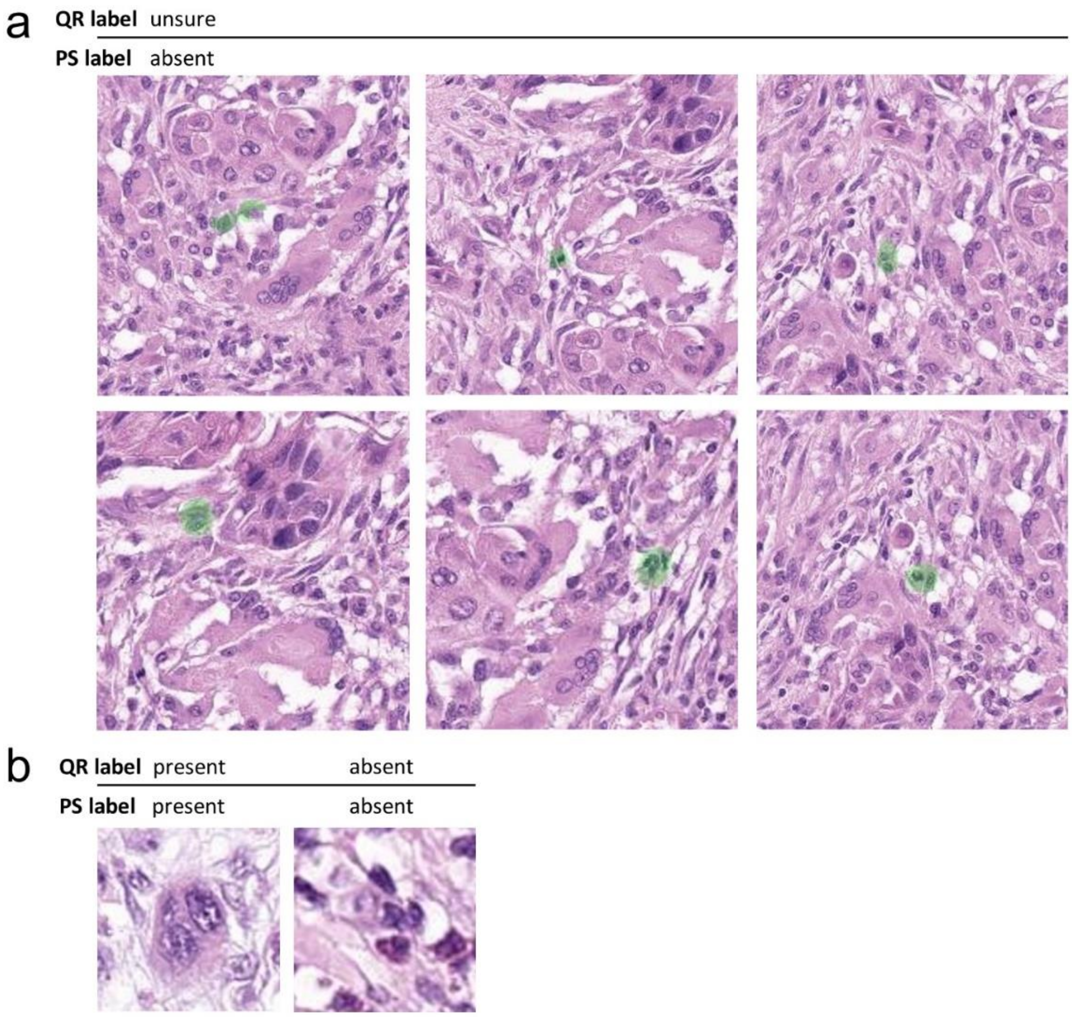

Supplementary Figure 4. a Discordant cases between PS and QR in the lung tumor budding use-case with u-net generated segmentation mask indicating tumor-bud candidate position (green). This overlay was necessary as multiple tumor bud candidates may be present per ROI. All discordant cases shown were labeled as unsure in QR and absent in PS. QR, as opposed to PS, presents a ROI rather than a patch, making context immediately available to the user. Discussion of discordant patches with the pathologist conducting the experiment indicates that the availability of context led the pathologist to be more doubtful in labeling patches as 'absent'. In PS however, proximity of the patch in the embedding space to other 'absent' patches led the pathologist to label these patches as 'absent'. **b** Reference examples showcasing the tumor bud present and tumor bud absent phenotypes labeled in the use case.

Supplementary Figure 5. Discordant cases in renal tubular classification

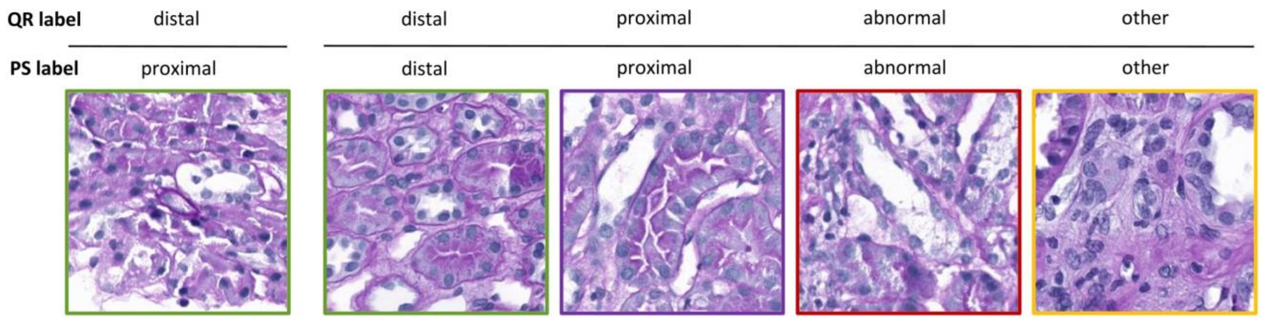

Supplementary Figure 5. Discordant case between PS and QR in the renal tubule labeling use case (left) and four examples of tubules per labeled class (right), with color indicating QR labels: distal (green), proximal (purple), abnormal (red) and other (e.g., non-tubule) (yellow). Discordant case between PS and QR was labeled as proximal in PS, while labeled distal in QR. The discordant image contains a small proximal tubule in the center and a distal tubule just off-center. It is therefore likely that during labeling efforts this led to confusion on which object to label.

Supplementary Figure 6. Discordant cases in renal glomerular classification

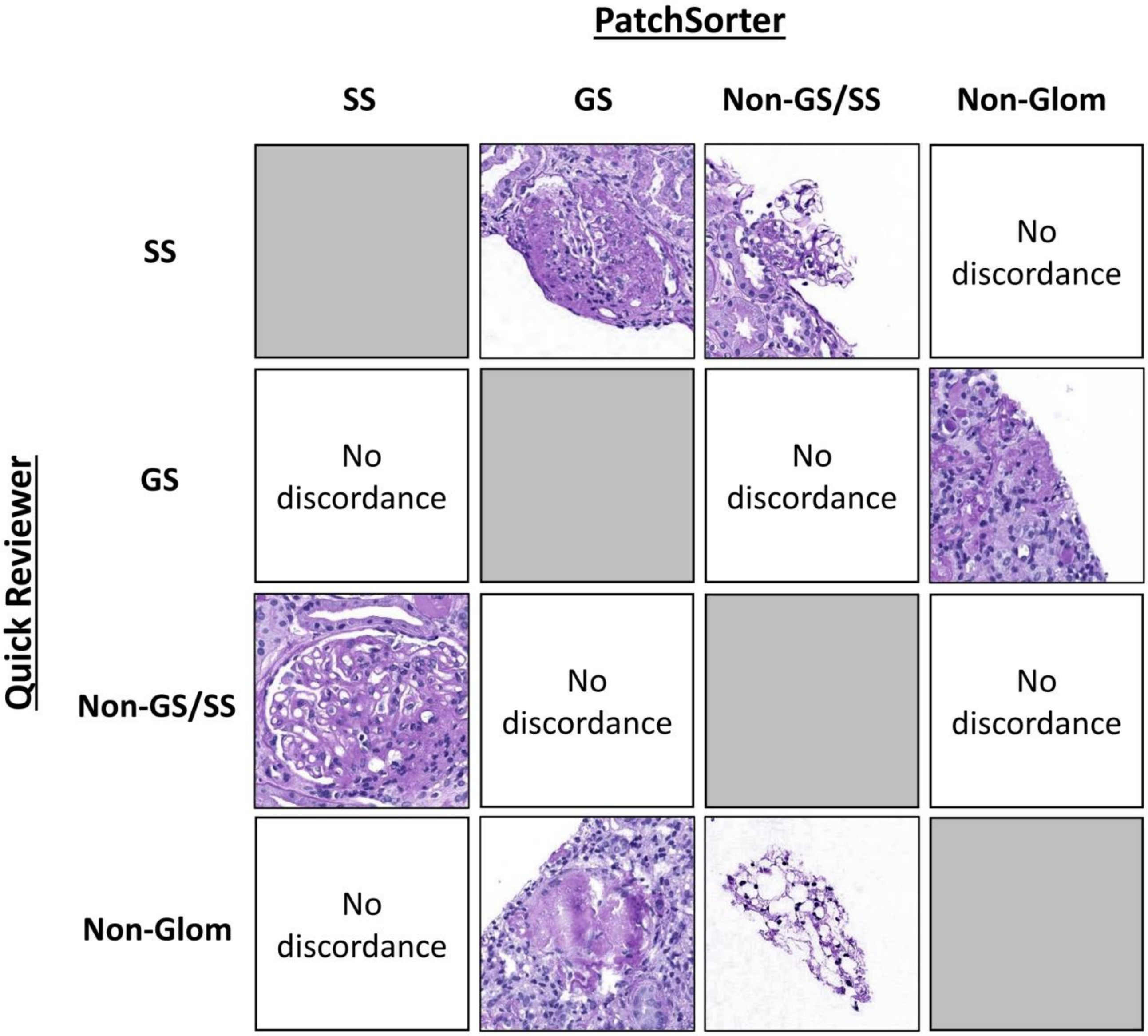

Supplementary Figure 6. Discordant cases between PS and QR in the glomerular labeling use-case. The structural complexity of the glomeruli and the high heterogeneity in SS and GS morphology, are likely contributing to the discordance between PS and QR. For example, a near to globally scarred glomerulus was labeled as SS using PS and vice versa (third panel from right). Similarly, a dense area of tissue is labeled GS on QR and non-glom on PS, and a glomerulus with a small area of sclerosis is labeled SS on QR and non-GS/SS on PS. Other examples of discordance labeling include 1) a glomerulus labeled non-GS/SS on QR and SS on PS: in this case the solidified area was interpreted as the vascular pole in one case and as sclerosis in the other; 2) a hyaline cast was labeled non-glom by QR but mistakenly labeled as GS on PS; 3) a floating fragment of non-better identifiable tissue (non-Glom by QR) is mistaken for non-GS/SS on PS.
